# Regulation of peripheral glucose levels during human sleep

**DOI:** 10.1093/sleep/zsaf042

**Published:** 2025-02-23

**Authors:** Xuefeng Yang, Fernando Tavares Fedumenti, Niels Niethard, Manfred Hallschmid, Jan Born, Karsten Rauss

**Affiliations:** Institute of Medical Psychology and Behavioral Neurobiology, University of Tübingen, Tübingen, Germany; Graduate School of Neural and Behavioural Science, International Max Planck Research School, Tübingen, Germany; Institute of Medical Psychology and Behavioral Neurobiology, University of Tübingen, Tübingen, Germany; Institute of Medical Psychology and Behavioral Neurobiology, University of Tübingen, Tübingen, Germany; Department of Cognitive Sciences, University of California, Irvine, Irvine, CA, USA; Institute of Medical Psychology and Behavioral Neurobiology, University of Tübingen, Tübingen, Germany; German Center for Diabetes Research (DZD), Tübingen, Germany; Institute for Diabetes Research and Metabolic Diseases of the Helmholtz Center Munich at the University of Tübingen (IDM), Tübingen, Germany; German Center for Mental Health (DZPG), Tübingen, Germany; Institute of Medical Psychology and Behavioral Neurobiology, University of Tübingen, Tübingen, Germany; German Center for Diabetes Research (DZD), Tübingen, Germany; Institute for Diabetes Research and Metabolic Diseases of the Helmholtz Center Munich at the University of Tübingen (IDM), Tübingen, Germany; German Center for Mental Health (DZPG), Tübingen, Germany; Werner Reichardt Center for Integrative Neuroscience, University of Tübingen, Tübingen, Germany; Institute of Medical Psychology and Behavioral Neurobiology, University of Tübingen, Tübingen, Germany

**Keywords:** sleep spindle, slow waves, neuroendocrinology, diabetes

## Abstract

Studies in rats indicate that oscillatory signatures of memory processing during sleep, specifically hippocampal sharp wave-ripples, also regulate peripheral glucose concentration. Here, we examined whether there is a similar link between such signatures of memory processing and glucose regulation during sleep in healthy humans. We obtained polysomnographic recordings and continuous recordings of peripheral interstitial glucose levels (1 sample/minute) from 10 participants (5 females) during two consecutive nights. Temporal relationships between electroencephalography **(**EEG) events of interest and glucose levels were examined using cross-correlation functions and peri-event time histograms. Confirming the findings in rats, we found that sleep spindles, a core signature of sleep-dependent memory processing, were followed within 1–6 minutes by a robust decrease in glucose concentrations. By contrast, slow oscillation events hallmarking slow wave sleep were followed, with a lag of 5–11 minutes, by an increase in glucose levels. Transitions into rapid eye movement sleep were followed by a glucose decrease after 10–14 minutes, whereas awakenings and microarousals were linked to immediate glucose increases. These temporal relationships indicate a sleep-specific regulation of peripheral glucose concentrations that is linked to both signatures of sleep-dependent memory processing as well as the macro-architecture of sleep. They possibly reflect noradrenergic regulation of sympathetic activity via the brainstem locus coeruleus and may be of relevance in clinical conditions with concurrent disturbances of sleep and glucose regulation.

## Graphical Abstract

**Graphical Abstract:**
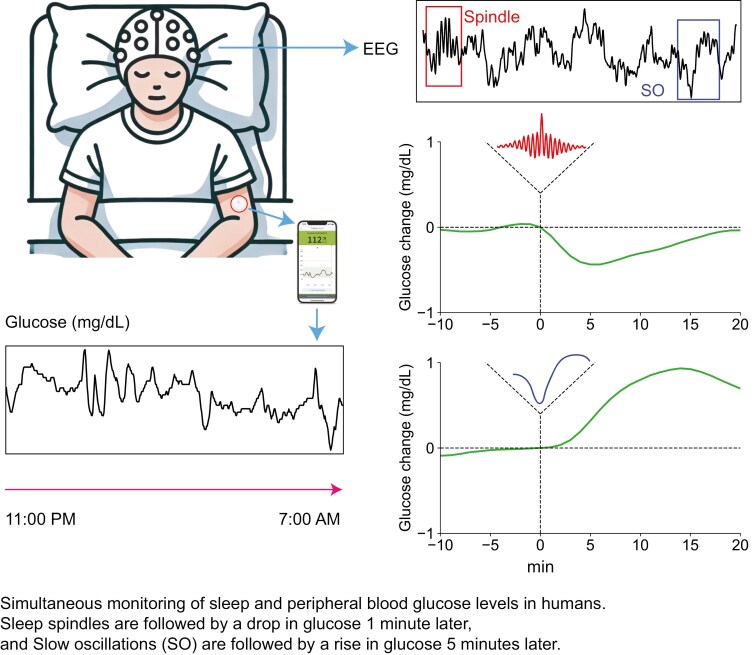

**Statement of Significance**

This study reveals a novel connection between sleep and glucose regulation in humans. By analyzing brain activity and glucose levels during sleep, we found that specific sleep events like sleep spindles and slow oscillations (SOs) trigger distinct glucose responses: sleep spindles, important for memory processing, were followed by a decrease in glucose, while SOs were linked to an increase. Sleep stage transitions also affected glucose, with increases during transitions to N1, N2, wakefulness, and microarousals, and decreases during REM. These findings suggest that sleep not only supports memory but also actively regulates metabolic balance, potentially through brainstem pathways. Understanding this connection could inform treatments for conditions where both sleep and glucose regulation are disrupted, such as diabetes and sleep disorders.

## Introduction

Sleep is not merely a period of rest and recovery, but a multifaceted dynamic process maintaining body and brain in an internal balance. The sleeping brain engages in the consolidation of memories acquired during the day, which in particular happens during periods of slow wave sleep (SWS) [1–4]. Simultaneously, the sleeping brain supports metabolic homeostasis, likely including an active regulation of peripheral glucose levels that is fine-tuned to the energy demands of both body and brain [5, 6]. However, how the memory processing interlinks with metabolism and, specifically, with glucose regulation during sleep is presently not clear.

Multiple observations indicate a robust link between sleep and metabolism on a macroscale, reflected by the close association of poor sleep quality, including short sleep duration, disturbed sleep, and irregular sleep patterns with blood glucose dysregulation [6–12]. Dysregulation of glucose concentrations appeared to be predominantly associated with non-rapid eye movement (NonREM) sleep rather than rapid eye movement (REM) sleep, inasmuch as the suppression of SWS in healthy adults reduced glucose tolerance and insulin sensitivity [13–15]. Only recently, attempts have been made to link the regulation of peripheral glucose concentrations to the micro-architecture of sleep, and specifically to electroencephalography (EEG) oscillatory signatures of memory processing during sleep. The most important of these oscillatory events are the neocortical slow oscillations (SOs), the thalamically generated sleep spindles, and the hippocampal sharp wave-ripples, which characterize human NonREM sleep and are thought to mediate a systems consolidation process underlying the strengthening of memory during sleep [4, 16–18]. Of central importance to the present study are findings in rats indicating that clusters of sharp wave-ripples recorded from the hippocampus are followed by a decrease in peripheral glucose concentrations within about 10 minutes [19]. This dip in peripheral glucose concentration could be also produced by optogenetically induced ripples in the hippocampus and prevented by suppressing lateral septum activity, altogether pointing towards a causal role of sleep-associated hippocampal ripple activity in the regulation of peripheral glucose levels.

The present study aimed at translating these findings from rats to healthy humans. As in rats, ripples in humans represent a core signature of memory processing accompanying the neuronal replay of newly encoded memories during NonREM sleep [20, 21]. However, originating from hippocampal networks, ripples currently cannot be directly assessed using surface EEG (or magnetoencephalography [MEG]) recordings in healthy humans. Effective memory processing during sleep, however, is not determined by the occurrence of ripples in isolation but is thought to depend essentially on the joint occurrence of ripples in hippocampal networks in the presence of spindles and SOs in thalamocortical networks [18, 22]. Hence, we focused on the link between peripheral glucose concentrations and spindles as well as SOs, as major signatures of sleep-dependent memory processing that likewise go along with memory replay and can be directly recorded on the surface EEG [18, 23, 24]. Indeed, the first findings in humans have suggested that the coupling between SOs and spindles during nocturnal sleep contributes to glucose homeostasis [25].

While there are first hints from experiments in rodents at a fine-tuned temporal link between signatures of memory processing during sleep and the regulation of peripheral glucose concentrations, knowledge about such a link in humans is missing. To address this gap, we continuously tracked peripheral interstitial glucose concentrations concurrently with polysomnographic signals during sleep in healthy humans. Our main finding is that sleep spindles in humans, like hippocampal ripples in rats, are followed (within about 5 minutes) by a decrease in peripheral glucose concentrations, supporting the view that the function of these oscillatory events in memory processing goes along with the fine-tuned regulation of metabolic processes.

## Materials and Methods

### Participants

Twelve healthy, nonsmoking participants took part in the experiment. Data from two participants were excluded due to poor EEG quality (over 50% noisy recordings because of technical problems). Thus, data from 10 participants (5 females) were retained for further analyses (mean age ± SD: 26.18 ± 3.07 years; range: 21–31 years). Routine examination ensured that they had a regular sleep-wake cycle, normal sleep quality (Pittsburgh Sleep Quality Index, PSQI ≤ 6), and were normal-weight (body mass index [BMI]: 20.83 ± 1.92 kg/m^2^, range 19.07–23.05 kg/m^2^). None of the participants reported neurological or psychological impairments or sleep disorders, and none had taken psychoactive medication during the previous 8 weeks, or had been on shift-work in the previous 4 weeks. All participants gave written informed consent and were paid for their participation. The study was approved by the Ethics Committee of the Medical Faculty of the University of Tübingen and adhered to established protocols and guidelines including the Declaration of Helsinki.

### Procedure and design

Prior to enrollment, participants completed an online prescreening for chronic medical conditions, current or previous mental illness, and neurological or sleep disorders. A telephone screening was then conducted to explain the procedure and answer any open questions. Participants were instructed to maintain a regular sleep-wake schedule for at least 4 weeks prior to the study (which in all cases meant going to bed between 10:00 pm and 01:00 am and to get up between 6:00 am and 9:00 am after a minimum of 6.5 hours of bedtime); to abstain from alcohol and caffeine, and to avoid excessive consumption of sugar during the 2 days before the experiment. They were also instructed to avoid excessive physical activity and to maintain their regular sleep habits.

One day prior to sleep recordings, participants completed questionnaires on demographics, health status, sleep habits, sleep quality, and dietary habits (not reported here). In addition, a continuous glucose monitor (FreeStyle Libre 3, Abbott, Chicago) was applied to the back of the participant’s nondominant arm to record glucose levels to ensure that all participants spent an adaptation night with the glucose meter. Participants were also instructed not to take vitamin C supplements during the study period as it is known to interfere with the FreeStyle Libre system [26].

Each participant underwent two consecutive nights of sleep recordings with concurrent glucose assessment. Participants were instructed not to nap during the day before the first night of recording, to eat their regular dinner at home, and to arrive at the laboratory at 9:45 pm. The content and caloric intake of the participants’ last meal before the overnight sleep session were not standardized, but participants were instructed to complete this dinner before 6:00 pm, i.e. 5 hours before sleep recordings started, to prevent any acute effects of food intake on glucose concentrations during the sleep period. After participants were prepared for polysomnographic recordings, their sleepiness, hunger, mood, and fatigue were assessed. Recording in the sleep lab began at 11:00 pm (lights off) and ended at 7:00 am (lights on). After waking up in the morning, participants filled in a sleep diary. They were instructed to spend the intermittent day as usual but to avoid strenuous exercise and again have dinner at home before 6:00 pm. The schedule of the second night of sleep recording was identical to that of the first night. The glucose meter was removed after the second recording night.

### EEG recordings and polysomnography

EEG signals were recorded from nine electrode sites (international 10–20 system locations, frontal—F3, Fz, and F4, central—C3, Cz, and C4, and parietal—P3, Pz, and P4) referenced to the average potential of electrodes placed at the mastoids (M1, M2), using a BrainAmp DC amplifier (Brain Products GmbH, Munich, Germany). The ground electrode was placed on the forehead. Vertical and horizontal eye movements (VEOG, HEOG) were recorded from electrodes above the right eye and below the left eye, and the electromyogram (EMG) was recorded from two electrodes placed on the chin. Ag-AgCl electrodes were used, and impedances were kept below 5 kΩ. All signals were sampled at 500 Hz. Signals were filtered (EEG and electrooculography [EOG] between 0.3 and 30 Hz, EMG with a high-pass of 5 Hz) and stored for offline analyses.

### Continuous glucose measurements

The Abbott FreeStyle Libre 3 Glucose Monitoring System for patients with diabetes was used for monitoring glucose concentration in the interstitial fluid (ISF). Compared to capillary glucose testing, the device yields a mean average relative difference of ~7.9% in patients with type 1 and type 2 diabetes over 6 years old. The device, which weighs 5 g, was placed on the back of the participant’s nondominant upper arm and secured to the skin with a Tegaderm dressing. It provides real-time glucose measurements between 40 and 400 mg/dL at a rate of one sample per minute. The device uses an amperometric method with an enzyme-coated electrode, whereby the enzyme glucose oxidase converts glucose into gluconic acid and hydrogen peroxide. The hydrogen peroxide is measured electrochemically with current values proportional to the glucose concentration. The sensitivity range of the glucose monitoring technique, based on the extraction of ISF, is between 0.09 and 1.08 mg/dL [27, 28]. The sensitivity of the glucose monitoring system used in the study was between 70.9 and 92.3% for glucose concentrations between 60 and 90 mg/dL [29]. The sensor was connected to a transmitter that sent real-time glucose measurements to a smartphone app.

### Sleep scoring and analyses

Sleep scoring was performed offline for 30-second epochs by two experienced scorers following the criteria outlined in the American Academy of Sleep Medicine (AASM) Manual for the scoring of sleep [30]. For each of the two recording nights, the following parameters were determined: total sleep time (TST, total duration of N1 + N2 + N3 + REM sleep), onsets and durations of individual sleep stages (N1, N2, SWS, and REM sleep), minutes awake after sleep onset (WASO), and sleep efficiency, i.e. the percent TST of time spent in bed (TIB).

For further analyses, EEG data were thoroughly inspected for artifacts, and 30-second epochs containing any artifacts were excluded. Also, channels with globally poor signal quality were omitted from subsequent analyses which happened in three nights (altogether 4 channels). Data from all channels were then down-sampled to 100 Hz (using MNE Python). Extraction of sleep spindles and SOs from N2 and SWS was performed separately for each night and channel using an algorithm adapted from the YASA-Python toolbox (Yet Another Spindle Algorithm) [31, 32]. In brief, for automatic spindle detection, the broadband EEG signal (1–30 Hz) was filtered in the sigma-band between 10 and 15 Hz, using zero phase-shift finite impulse response (FIR) filtering. Then, the root mean square (RMS) of the sigma-band signal was formed, based on a moving window of 300 ms (step-size of 100 ms) and the relative sigma power, i.e. the ratio of sigma power to 0.3–30 Hz broadband power was calculated, based on a Fourier transform of 2-second epochs with an overlap of 200 ms. Finally, the Pearson correlation coefficient between the broadband and sigma-band signals was obtained using a sliding window of 300 ms with a step-size of 100 ms. Potential spindle events were detected, separately in each electrode, whenever three thresholds were crossed at the same time: (a) sigma-band RMS exceeded mean RMS + 1.5 standard deviations of the RMS for the respective channel, (b) relative sigma power ≥0.2—i.e. the algorithm ensured that increased sigma-band activity was not driven by broadband power increases, and (c) the correlation between sigma-band and broadband activity was r ≥ 0.65—i.e. the algorithm assessed whether oscillations in the sigma-band were accompanied by broadband signal fluctuations of similar polarity, ensuring that the detected events were visible in the broadband signal commonly used for visual scoring [33]. Candidate spindles were then refined by smoothing the decision vector (i.e. the sum of parameters which crossed their respective thresholds) with a 100 ms window, merging events that were less than 500 ms apart, and rejecting those shorter than 0.5 second or longer than 2 seconds. Finally, windowed detection signals were resampled to the original time vector using cubic interpolation to improve temporal resolution.

For the YASA-based detection of SOs, data from N2 and SWS epochs were filtered between 0.3 and 1.5 Hz (FIR filter) and all zero-crossings were determined in the filtered signal. Candidate SOs were identified as negative peaks with an amplitude ranging from −40 to −200 µV, followed by positive peaks with amplitudes ranging from 10 to 150 µV. Events were retained as true SOs if their negative half-wave duration was between 0.3 and 1.5 seconds, their positive half-wave duration was between 0.1 and 1 second [34], and their peak-to-peak amplitude was between 75 and 350 µV. The zero-crossing preceding the negative peak was defined as SO onset. Coupled SO-spindle events were identified (using in-house scripts) as those SOs for which the peak of a spindle occurred within a ±1.2-second window around the negative SO peak [35].

Microarousals were automatically detected using an established algorithm based on AASM criteria [36, 37]. The algorithm identified transient microarousal events using EEG data from the Cz channel (in one case Fz was used because of artifacts in Cz) and EMG data that lasted more than 3 seconds and less than 15 seconds, with at least 10 seconds of preceding stable sleep. To identify microarousals, a short-time Fourier Transform (STFT; 3-second windows, step-size 0.2 second) was applied to the EEG signal to estimate power in the alpha band (8–12 Hz) and for frequencies >16 Hz. Baseline power was derived as the average across the 10 preceding seconds, and candidate events were identified when α power exceeded 2.5 times the baseline, or when power >16 Hz exceeded twice the baseline. The detected arousal events were then refined using adaptive thresholding of EEG and EMG amplitudes, and spindle events were excluded. During REM sleep, identification of a concurrent increase in EMG amplitude for at least 1 second was required for events to be retained as microarousals [38]. EMG signals were high-pass filtered (cutoff 15 Hz) to remove low frequencies unrelated to muscle activity, and EMG amplitudes during candidate events were calculated and compared to an average reference amplitude surrounding the event (15 seconds before the start of the event to 15 seconds after the end of the event) and marked as arousal events if they exceeded 1.4 times the reference value.

### Statistics

All analyses were performed using standard Python (scipy.stats) and R (lme, emmeans) functions and libraries [39]. Time series of glucose and EEG data were synchronized via Unix timestamps, and sleep parameters were calculated for 1-minute time bins starting from “lights off” (11:00 pm). Cross-correlation functions were calculated between the time series of spindle, SO, and coupled SO-spindle events/minute across all channels and the first derivative (i.e. the instantaneous change) of glucose concentration values [19]. Cross-correlation coefficients were transformed into Fisher’s *Z*-values and then averaged across all 18 recording nights (i.e. two consecutive nights per participant; data from two nights were excluded because of sleep efficiency <80%). Data for each time lag were tested against zero using one-sample *t*-tests. No corrections were applied for multiple comparisons given the exploratory nature of this analysis and the limited number of time lags tested. However, effects were considered significant only if they were stable across at least two consecutive time points (i.e. for 2 minutes). Indeed, temporal clustering of the effects reported below suggests that our findings are unlikely to have arisen by chance, as type-I errors would be randomly distributed under the null hypothesis. To ensure transparency, both uncorrected and Benjamini–Hochberg (BH) corrected p-values for key comparisons will be reported for time lags ranging from −20 to +20.

To further investigate the relationship between sleep events and glucose concentrations, we computed peri-event time histograms (bin size 1 minute), with the events of interest (spindle onsets, SO onsets, sleep stage transitions, etc.) serving as reference (t = 0 minute) while glucose concentrations were averaged in a −10 to 20-minute window around the reference event. Glucose concentration for each spindle, SO event, and coupled SO-spindle event was expressed as a difference value with reference to the level at t = 0 minute. For sleep stage transition events, glucose concentration was referenced to the mean value between −6 minutes and −1 minute as a more stable reference. Average peri-event histograms were calculated across all recording channels using a weighted average to account for variations in event frequency across nights and participants. Differences from baseline were assessed by pointwise comparisons using *t*-tests (against zero, uncorrected for multiple testing) to identify the time ranges with changes from baseline as indicated by significant differences for at least two consecutive time points (i.e. 2 minutes) [40, 41].

To examine the relationship of glucose concentrations with SOs and spindle events detected at different recording sites, we used a linear mixed-effects model with “recording site” as a fixed effect and participant as a random effect. Post hoc paired-samples *t*-tests, adjusted by the Tukey method, were then performed to identify significant differences between any two recording sites. Results were considered significant at p < 0.05. Data are presented as mean ± SEM.

### Results

Recordings indicated normal sleep architecture (Tables 1 and 2; for individual data, see Supplementary Table S1). Table 1 summarizes the parameters of sleep macro-architecture. Density values for spindles, SOs, and coupled SO-spindle events are shown in Table 2. Comparisons between the participants’ first and second experimental nights did not reveal any differences except that density of SOs (3.41 ± 0.56 vs. 4.04 ± 0.44, t = −2.54, and p = 0.039) and coupled SO-spindle events (2.23 ± 0.50 vs. 2.99 ± 0.48, t = −2.53, and p = 0.040) was slightly higher in the second night, suggesting a habituation effect across the two nights in the laboratory. Moreover, an exploratory analysis of cross-correlation functions between sleep parameters and glucose concentrations did not reveal any differences between the experimental nights. Accordingly, the analyses reported below are based on the collapsed dataset across the participants’ first and second experimental nights. Spectral analyses of the time course of power in the 12–16 Hz spindle band during NonREM sleep confirmed the presence of an infraslow rhythm underlying spindle activity with a mean cycle length of 55.56 ± 100 seconds (0.018 ± 0.01 Hz), whereas such a rhythm was not discernible for slow wave activity (SWA) (Supplementary Figure S1A, B) [42, 43].

**Table 1.** Summary of Sleep Characteristics

	Mean	SEM
TST (minute)	432.50	6.09
SOL (minute)	10.03	1.52
WASO (minute)	33.50	3.50
N1 (minute)	43.47	2.30
N2 (minute)	239.19	5.59
SWS (minute)	55.06	5.76
REM (minute)	94.78	4.25
Microarousal (counts)	37.28	5.59
SWS latency (minute)	28.33	4.15
REM sleep latency (minute)	94.83	8.52

Parameters of sleep architecture averaged across 18 nights. Data are mean (SEM). Total sleep time (TST), time spent in N1, N2, SWS and REM sleep (in minute). SOL: sleep onset latency. WASO: wake after sleep onset. Microarousals (counts) and latency (from sleep onset) of SWS and REM sleep are also indicated (in minute).

**Table 2.** Spindle, SO, Coupled SO-Spindle Density Across Recording Sites

	Spindle density	SO density	Coupled SO-spindle density
Frontal	1.59 (0.14)	4.60 (0.34)	3.62 (0.31)
Central	1.69 (0.16)	3.84 (0.32)	2.50 (0.23)
Parietal	2.25 (0.19)	3.37 (0.30)	2.11 (0.18)

Data are mean (SEM). *N* = 18 nights. Mean density (events per minute) in NonREM sleep (N2 and SWS) at frontal, central and parietal electrode sites are indicated.

### Glucose concentration decreases after sleep spindles

**Figure 1.**
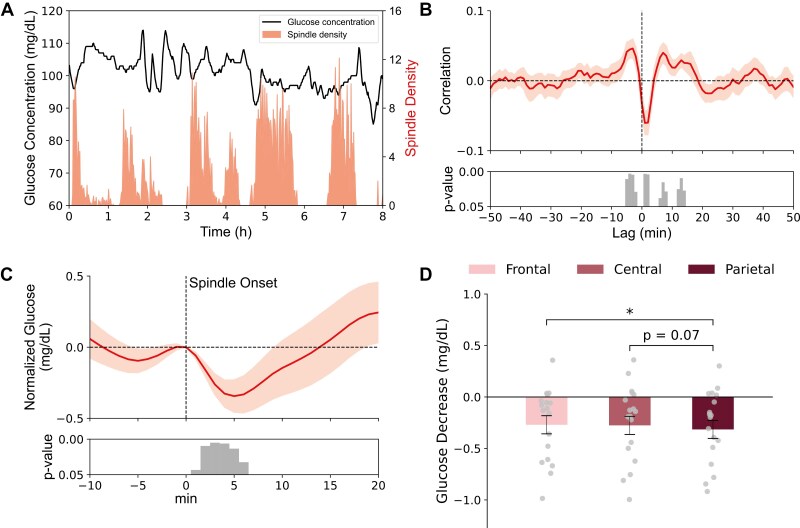
Spindles are followed by a decrease in glucose levels. (A) Example data from one night in an individual participant. Glucose levels plotted against spindle density (per minute). (B) The average cross-correlogram between spindle density and glucose concentration changes across all 18 nights for time lags between ±50 minutes. Note, negative peak correlation at a lag +1 minute indicates that spindle activity is followed by a decrease in glucose 1 minute later. Bottom panel indicates significance of correlation coefficients (one-sample *t*-test against 0). (C) Peri-event time histogram showing the average time course of glucose concentration −10 to +20 minutes around the onset of spindle events (0 minute) across all electrode sites and nights (*n* = 18, glucose level at 0 minute = 90.66 ± 0.03 mg/dL). Glucose concentrations are indicated as difference values with the concentration at 0 minute set to 0 mg/dL. Bottom panel indicates significance (one-sample *t*-test against zero) reaching a maximum between 1 and 6 minutes after spindle onset. (D) Mean ± SEM decreases in glucose concentration (1–6 minutes after spindle onset) for spindles detected in frontal, central, and parietal channels (dot plots overlaid); p-values for pairwise comparisons are indicated.

Cross-correlation functions between sleep spindle density (averaged across all recording channels) and glucose concentration changes revealed a negative peak correlation at a time lag of +1 minute, indicating that increased spindle activity is followed within about 1 minute by a decrease in glucose concentration (r = −0.06 ± 0.02; t[17] = −3.44, p = 0.003, and BH-corrected p = 0.046; Figure 1A, B). A negative peak correlation with lags between 0 and 2 minutes was similarly identified in the great majority (15 out of 18) of the individual cross-correlation functions calculated for each night. Notably, although lower in amplitude, there were also significant positive cross-correlation coefficients before (at a lag of about −3 minute) and after the prominent negative peak cross-correlation (lags between +6 and +14 minutes; Figure 1B). The decrease in glucose concentrations following spindles was likewise observed in the peri-event correlation histograms, which revealed a highly significant decrease in glucose concentration averaging ~0.24 mg/dL between 1 and 6 minutes following the onset of a spindle event (t[17] < −2.18, p < 0.05, for the 1–6 minutes post spindle onset interval; Figure 1C) which persisted up to 6 minutes after spindle onset (6 minutes, −0.33 ± 0.13 mg/dL, t[17] = −2.33, and p = 0.032). The decrease tended to be greater following parietal than frontal and central spindles (F[2, 16] = 3.83, p = 0.022, for the effect of recording channel; Figure 1D; Table 2).

### Glucose concentration increases following SOs

**Figure 2.**
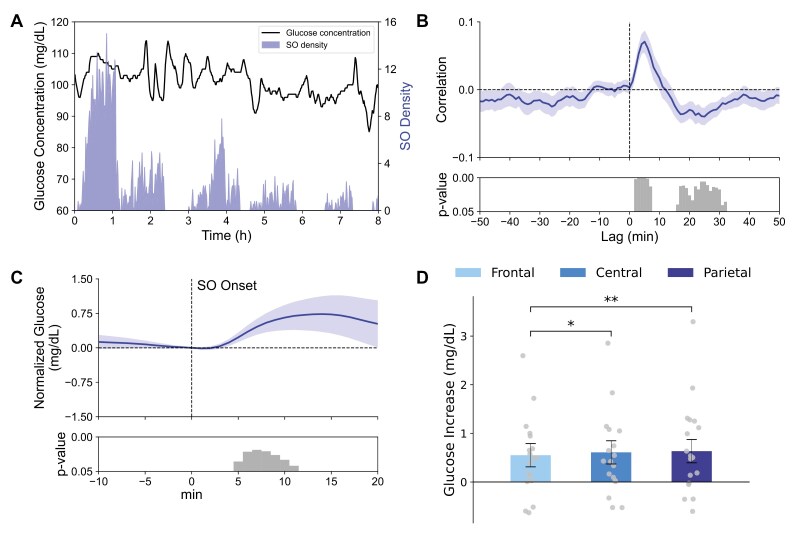
Slow oscillations (SOs) are followed by an increase in glucose concentrations. (A) Example data from one night in an individual participant. Glucose levels plotted against SO density (per minute). (B) Average cross-correlogram between glucose concentration change per minute and corresponding SO density reveals a distinct positive peak correlation at a lag of +5 minutes, indicating that increased SO activity is followed by an increase in glucose levels 5 minutes later. Bottom panel indicates significance of correlation coefficients (one-sample *t*-test against 0). (C) Peri-event time histogram showing the average time course of glucose concentration −10 to +20 minutes around the onset of SO events (0 minute) across all electrode sites and nights (*n* = 18, glucose level at 0 minute = 91.25 ± 0.03 mg/dL). Glucose concentrations are indicated as difference values with the concentration at 0 minute set to 0 mg/dL. Bottom panel indicates significance (one-sample *t*-test against zero) reaching a maximum between 5 and 11 minutes after SO onset. (D) Mean ± SEM increases in glucose concentration (5–11 minutes after SO onset) for SOs detected in frontal, central, and parietal channels (dot plots overlaid); p-values for pairwise comparisons are indicated.

Cross-correlation functions between SO density and glucose concentration changes revealed a positive peak, indicating that an increase in SO density was followed, with a time lag of +5 minutes, by an increase in glucose concentrations (r = 0.07 ± 0.02, t[17] = 4.47, and p < 0.001, BH-corrected p = 0.005; Figure 2A, B). This initial positive peak was followed by a negative peak correlation at a time lag of about 17 minutes, suggesting a relatively rapid downregulation of glucose concentrations following their initial increase (r = −0.04 ± 0.01; t[17] = −2.80, p = 0.012, and BH-corrected p = 0.066; Figure 2A, B). The peri-event time course of glucose concentrations (centered on the onsets of SO events) confirmed a transient increase in glucose concentration reaching significance between 5 and 11 minutes after SO onsets (0.51 ± 0.01 mg/dL, t[17] > 2.20, and p < 0.05, for the 5–11 minutes post-SO onset interval; Figure 2C). At the end of the 30-minute window, glucose concentrations had fully returned to baseline levels. The increase in glucose concentration was more pronounced following SOs detected at posterior recording sites (central, parietal) than following frontal SOs (F[2, 16] = 6.93, df = 2, p < 0.001, for effect of recording channel; Figure 2D; Table 2).

### Glucose increases following SOs override decreases following spindles

Considering that spindles and SO events were followed by opposite changes in glucose concentrations, we aimed at separating the relative contributions of spindles and SOs in explaining glucose level fluctuations. In the first approach, based on the outcomes of the cross-correlation functions between glucose concentration changes and spindle and SOs density, respectively, we calculated a forward stepwise regression model with spindle density at the time lag +1 minute and SO density at the time lag + 5 minutes as predictors, and glucose change (per minute) as the regressor. Results indicated that both spindle density and SO density significantly explained glucose change per minute (spindle: b = −0.03, t = −5.00, and p < 0.001; SO: b = 0.02, t = 5.26, and p < 0.001), suggesting a relatively independent contribution of spindle and SO activity to subsequent changes in glucose levels.

**Figure 3.**
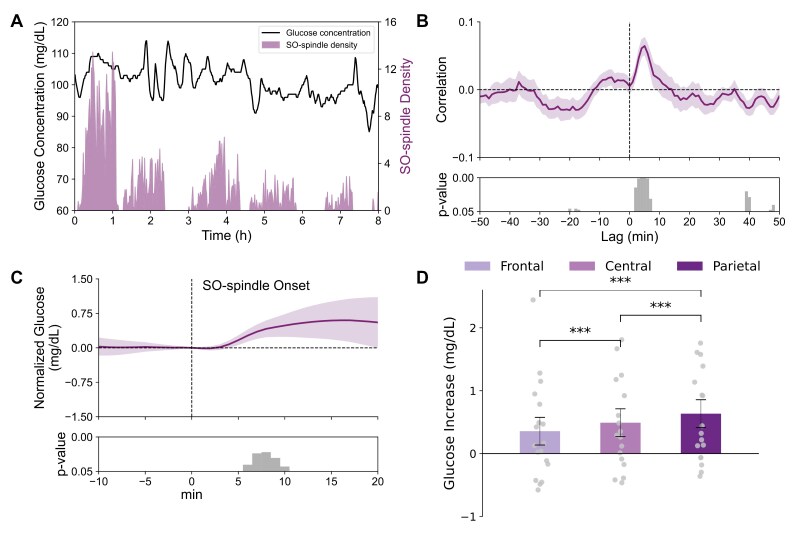
Coupled slow oscillation-spindle events are followed by an increase in glucose concentrations. (A) Example data from one night in an individual participant. Glucose levels plotted against SO-spindle event density (per minute). (B) Average cross-correlogram between glucose concentration change per minute and corresponding SO-spindle event density reveals a distinct positive peak correlation at a lag of +5 minutes, indicating that increased density of SO-spindle events is followed by an increase in glucose levels 5 minutes later. Bottom panel indicates significance of correlation coefficients (one-sampled *t*-test against 0). (C) Peri-event time histogram showing the average time course of glucose concentration −10 to +20 minutes around the onset of coupled SO-spindle events (0 minute) across all electrode sites and nights (*n* = 18, glucose level at 0 minute = 91.14 ± 0.04 mg/dL). Glucose concentrations are indicated as difference values with the concentration at 0 minute set to 0 mg/dL. Bottom panel indicates significance (one-sample *t*-test against zero) reaching a maximum between 6 and 10 minutes after SO-spindle event onset. (D) Mean ± SEM increases in glucose concentration (6–10 minutes after SO onset) for SOs detected in frontal, central, and parietal channels (dot plots overlaid), p-values for pairwise comparisons are indicated.

In a second approach, we focused on co-occurring spindle and SO events, i.e. SO-spindle events. Cross-correlation functions between SO-spindle event density and glucose concentration changes revealed a positive peak correlation at a lag of +5 minutes, indicating that such events are followed by an increase in glucose concentrations about 5 minutes later (r = 0.06 ± 0.01; t[17] = 5.03, p < 0.001, and BH-corrected p = 0.004; Figure 3A, B). The peri-event time course of glucose concentrations centered on the onset of SO-spindle events confirmed a significant increase in glucose concentration 6–10 minutes after SO-spindle events (0.39 ± 0.01 mg/dL, t[17] > 2.18, and p < 0.05; Figure 3C). This increase was more pronounced for parietal than frontal or central SO-spindle events (F[2, 16] = 37.24, p < 0.001; Figure 3D, Table 2). Finally, analyses of spindles occurring in isolation, i.e. in the absence of an SO, as well as of SOs occurring in isolation, i.e. in the absence of a spindle, confirmed the opposing changes in glucose concentration following these isolated spindles and SOs (Supplementary Figure S2).

### Transitions into sleep stages are associated with distinct changes in glucose concentrations

**Figure 4.**
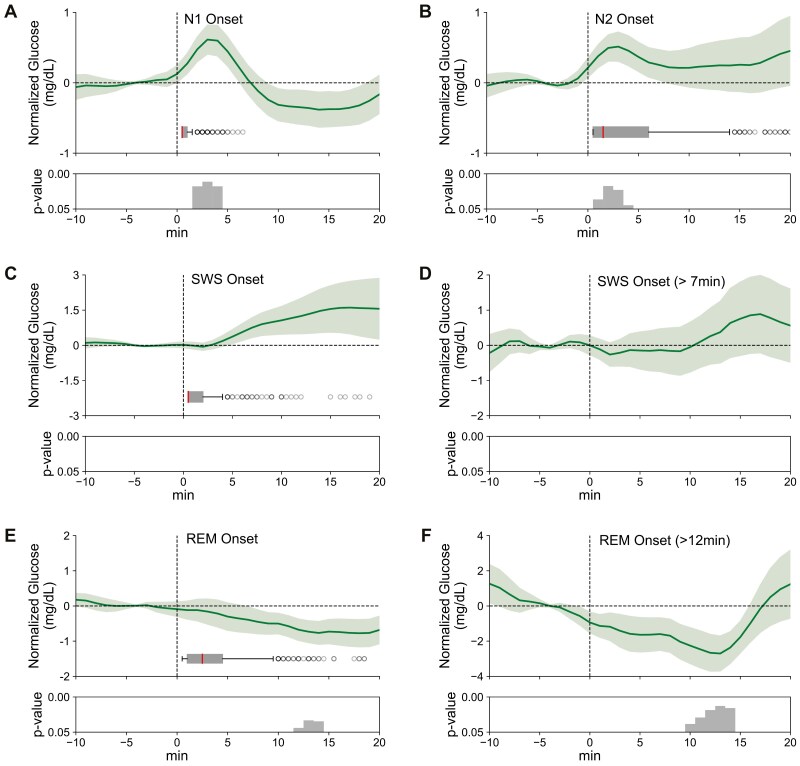
Glucose concentrations associated with transitions into different sleep stages. Peri-event time histogram showing the average time course (means ± SEM) of glucose concentrations around the onset (0 minute) of (A) N1 epochs (*n* = 18, across all nights, average duration 0.96 ± 0.03 minutes, glucose level at baseline = 91.04 ± 0.32 mg/dL), (B) N2 epochs (*n* = 18, average duration 5.20 ± 0.30 minutes, glucose level at baseline = 91.03 ± 0.31 mg/dL), (C) SWS epochs (*n* = 18, average duration 3.24 ± 0.35 minutes, glucose level at baseline = 90.49 ± 0.52 mg/dL), (D) SWS epochs longer than 7 minutes (*n* = 33, glucose level at baseline = 95.01 ± 1.97 mg/dL), (E) REM sleep epochs (*n* = 18, average duration 4.00 ± 0.35 minutes, glucose level at baseline = 90.96 ± 0.39 mg/dL), and (F) REM sleep epochs longer than 12 minutes (*n* = 24, glucose level at baseline = 91.64 ± 1.99 mg/dL). Glucose concentrations are shown for an interval starting 10 minutes before event onset and ending 20 minutes later. Horizontal gray boxplots indicate epoch duration (median is indicated by a vertical line, whiskers extend to 1.5 times the interquartile range). Concentrations are indicated as difference values with the concentration –6 to –1 minute set to 0 mg/dL. Bottom panels indicate significance (one-sample *t*-test against zero).

Glucose concentrations were also affected by sleep stage transitions. Transitions into N1 were associated with a transient increase in glucose levels which reached significance 2 minutes after the transition (0.48 ± 0.18 mg/dL, t[17] = 2.63, and p = 0.018, Figure 4A). Onsets of N2 were likewise accompanied by an increase in glucose concentration starting already 1 minute after the N2 onset (0.38 ± 0.16 mg/dL, t[17] = 2.27, and p = 0.036, Figure 4B). Changes in glucose concentration at transitions into SWS were not significant. There was a trend towards increased glucose concentrations 7–10 minutes after the onset of SWS epochs, i.e. a time when most SWS epochs had already ended (Figure 4C). As we suspected that this increase rather reflected the transition into a lighter sleep stage at the end of the SWS epoch, we performed an additional exploratory analysis focusing on SWS epochs longer than 7 minutes. (Note, as the number of such longer SWS epochs was rather small, statistical evaluation relied on the total number of epochs across all nights). This analysis confirmed the null-finding, i.e. there were no significant changes in glucose concentration after SWS onset (Figure 4D). Transitions into REM sleep were followed, with a delay of 12 minutes, by a decrease in glucose concentration lasting until 14 minutes (−2.58 ± 0.56 mg/dL, t[17] < −2.17, p < 0.05; Figure 4E). In light of the long delay of the decrease, we again performed an additional analysis focusing on REM epochs lasting longer than 12 minutes (Figure 4F). These analyses confirmed a significant decrease in glucose concentrations 10–14 minutes after REM sleep onset (−2.49 ± 0.44 mg/dL, t[23] < −2.19, and p < 0.04).

We performed supplementary cross-correlation analyses between changes in glucose concentrations and power in sleep EEG frequency bands that are characteristic of different sleep stages, like SWA, sigma band activity, and theta band activity (Supplementary Figure S3). The analyses basically confirmed the relationships with glucose concentrations, as reported here for SO and spindle events as well as REM sleep transitions.

### Increases in glucose concentration at transitions into wakefulness and microarousals

**Figure 5.**
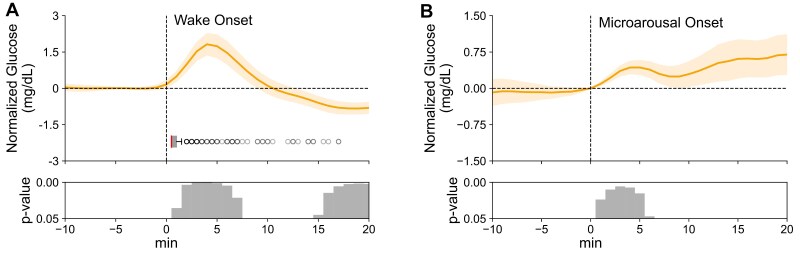
Peri-event time histograms showing the average time course (means ± SEM) of glucose concentrations (A) around the onset (0 minute) of wake epochs (*n* = 18, summed across all nights, average duration 1.79 ± 0.78 minutes, glucose level at baseline = 90.89 ± 0.36 mg/dL) and (B) around microarousals (*n* = 18, minute, glucose level at 0 minute = 91.29 ± 0.36 mg/dL). Glucose concentrations are shown in a window ±15 minutes around the target event and indicated as difference values with the concentration –6 to –1 minute set to 0 mg/dL. Horizontal gray boxplots indicate epoch duration (median is indicated by a vertical line, whiskers extend to 1.5 times the interquartile range). Bottom panels indicate significance (one-sampled *t*-test against zero).

Whenever the participants entered periods of wakefulness, glucose concentrations began to rise distinctly in the first minute following wake onset (concentration at 1 minute: 0.49 ± 0.21 mg/dL, t[17] = 2.28, and p = 0.036) with the peak increase of 1.82 ± 0.44 mg/dL reached about 4 minutes after wake onset, i.e. a time when the average wake episode had already ceased (Figure 5A). Notably, a similar increase in glucose concentration was observed following microarousals, reaching a maximum 4–5 minutes after the microarousal (mean increase in glucose concentration during this interval 0.43 ± 0.13 mg/dL, t[17] > 2.69, and p < 0.02; Figure 5B).

### Discussion

Our results indicate an association between peripheral glucose concentrations and sleep macro- and micro-architecture on a timescale of several minutes. Cross-correlation functions and peri-event time histograms consistently indicated that sleep spindles are followed by a decrease in peripheral glucose concentrations within 1 minute, with minimum levels reached after 1–6 minutes. SOs, on the other hand, were followed by increases in peripheral glucose concentrations that developed more slowly, reaching maximum levels between 5 and 11 minutes. Results for coupled SO-spindle events suggest that the enhancing effect of SOs overrides the downregulating effect of spindles on peripheral glucose levels. In terms of sleep architecture, transitions into N1 sleep, N2 sleep, and wakefulness, as well as microarousals, were followed by distinct increases in glucose levels. No significant glucose changes were seen after the onset of SWS, but REM sleep was associated with a significant decrease in glucose concentrations. Collectively, these findings indicate that peripheral glucose concentrations are subject to a fine-tuned regulation by sleep, and specifically by sleep spindles and SOs, i.e. oscillatory signatures well-known to support memory processing during sleep.

Our experiment was based on evidence in rats indicating that increased hippocampal ripple activity—another sign of memory processing during sleep—is followed by a decrease in peripheral glucose levels, and that this decrease is mediated via the lateral septum and a top-down hypothalamic signaling pathway [19]. Hippocampal ripples are often coupled to the excitatory troughs of spindles originating from the reticular nucleus of the thalamus [17, 18, 44]. Accordingly, spindles, like hippocampal ripples, tend to occur together with memory reactivations in hippocampal and cortical areas [18, 45]. Given that hippocampal ripples cannot be reliably measured in healthy volunteers, we relied on spindles as the noninvasive measure most closely related to memory reactivations to assess the link between sleep-dependent memory processing and peripheral glucose levels in humans. Our finding of a systematic decrease in glucose concentration emerging within less than 5 minutes after spindle events corroborates the findings of Tingley et al [19], but using an alternative index of memory reactivation in healthy humans. The fact that this decrease was seen earlier (after about 1 minute) in our participants than in rats (after about 10 minutes) may be owed to the higher glucose sampling rate in the present experiment compared to Tingley et al [19]. Also, the size of the effect in this rat study (with peak cross-correlation coefficients of r = −0.21 between ripple density and glucose concentrations) is roughly comparable, although slightly higher than that observed here in humans, which may be due to the higher signal-to-noise ratios that can be achieved by invasive glucose monitoring in rats, but which could also indicate a stronger link of hippocampal ripples than EEG spindles to the mechanism mediating the decrease in glucose concentration.

Surprisingly, SOs—which are also involved in memory reactivations in the hippocampus-dependent episodic memory system [4, 16]—were not followed by a decrease but by a distinct increase in peripheral glucose levels, an effect that emerged more slowly than the spindle-related decreases. Given that there are different subtypes of slow waves with highly overlapping frequency characteristics but opposing functions in memory consolidation [46, 47], it might be argued that our assessment of SOs was too crude. However, separate analyses of only those SOs that were coupled with spindles showed that these nesting events—which most closely reflect memory reactivations—were also followed by an increase in glucose concentrations. The opposite effects of SOs and spindles on glucose levels match with data from human studies showing that slow oscillatory EEG activity is less consistently linked to memory improvements than spindles [48]. In fact, some studies have reported negative correlations between measures of slow oscillatory EEG activity and the retention of memory [49]. Whatever the exact role of SOs in memory processing, the present findings of distinct changes in peripheral glucose levels following SOs suggest that they serve functions different from those of thalamic spindles and hippocampal ripples. The findings on coupled SO-spindle events moreover suggest that when spindles and SOs synergize in memory processing, the enhancing effects on glucose levels accompanying SO-events override the decrease in glucose levels following spindles.

The opposing relationships of sleep spindles and SOs with peripheral glucose levels did not translate into corresponding global differences between sleep stages dominated by the different classes of events: transitions into N2 were associated with increased glucose levels, whereas no significant changes were observed following the onset of SWS. This suggests that the global state of the brain and body exerts sleep stage specific effects on peripheral glucose regulation that may even surpass the accumulated effects of spindles and SOs preferentially occurring during N2 and SWS, respectively. Intriguingly, transitions into REM sleep were associated with a decrease in peripheral glucose levels at substantially longer delays (>10 minutes), and this effect was more pronounced when focusing on extended REM periods (>12 minutes).

We can only speculate about the mechanisms mediating the observed changes in glucose levels following sleep oscillatory activity and sleep stage transitions. In principle, changes in peripheral glucose concentrations may represent a kind of passive entrainment to the brain’s energy consumption where gross increases in neuronal firing activity (and energy consumption) lead to a transient drop in peripheral glucose concentrations and, vice versa, decreases in the brain’s energy consumption produce a transient increase in glucose concentrations in the periphery [5, 50]. If this were the case, overall reductions of cortical firing rates during SWS [51, 52] would be expected to entail increases in peripheral glucose levels. However, our data show that changes in peripheral glucose following SWS onset are negligible. Conversely, for REM sleep, which is assumed to go along with increased neural activity, passive entrainment would suggest a dip in peripheral glucose levels, in accordance with our findings.

Beyond passive entrainment, the brain actively regulates peripheral glucose concentrations in a top-down fashion, mainly via two pathways: (a) by the release of glucocorticoids via the hypothalamus-pituitary-adrenal (HPA) axis; and (b) via autonomic nervous system efferences. Regulation via the HPA is too slow to explain changes emerging within a few minutes after sleep spindles and SOs [53, 54]. However, the pattern of changes in glucose levels we report agrees with the view that during sleep, the brain regulates peripheral glucose levels via autonomous nervous system efferences, with the locus coeruleus (LC) as a hub coordinating central and peripheral aspects of noradrenergic regulation [6, 55]. Thus, activation of noradrenergic LC projections targeting both sympathetic and parasympathetic autonomic pathways can lead to short-term increases in blood glucose levels within less than 30 minutes [56, 57].

At the same time, the LC is involved in the regulation of SOs, spindles, and hippocampal ripples: LC activity preferentially occurs during the rising phase of SOs [58, 59], and there is evidence that LC activity contributes to the termination of spindle activity and impairs the coupling of hippocampal ripples with spindles [60–62]. Thus, coordinated spindle and ripple activity preferentially occurring during periods of low LC activity could explain the decrease in peripheral glucose concentration following spindles in humans (in the present study) and, respectively, following ripples in rats [19]. Likewise, LC activity coupled to the SO upstate fits the enhancement of peripheral glucose concentrations systematically following SOs. Finally, REM sleep is associated with a suppression of LC activity [61, 63], matching our observation that peripheral glucose concentrations decline after participants enter REM sleep. The increases in peripheral glucose seen after awakenings, and to a lesser degree, after microarousals, are also likely to reflect concomitant increases in noradrenergic LC activity [63–69]. However, although the pattern of changes in glucose concentrations agrees with the view of LC as a key mediator of these changes, this hypothesis remains to be directly tested.

A further issue to be addressed in future studies concerns the presence of a feedback loop in the brain’s regulation of glucose levels during sleep. Our cross-correlation functions suggest that the decrease in glucose levels after spindles is systematically followed, within 15 minutes, by an increase of similar amplitude and longer duration. The cross-correlogram for SOs likewise indicates such counter-regulation, with the increase in glucose levels emerging after SOs followed by decreases within about 17 minutes. Feedback responses based on peripheral glucose concentrations have been reported at roughly comparable delays of ~25 minutes, although glucose-sensitive neurons in the hypothalamus respond to physiological fluctuations in blood glucose at a distinctly faster rate of 60 to 90 seconds [70, 71]. The existence and nature of such feedback effects thus remain to be established in further experiments.

One limitation of our study is that the continuous glucose monitors assess interstitial glucose concentrations, as opposed to blood glucose levels. Given the time lags associated with the diffusion of glucose from blood to interstitial fluid and between interstitial glucose changes and sensor responses [72, 73], this approach limits the temporal precision of linking neural events to glucose changes. Experiments in healthy adults (in the overnight fasted state) revealed delays in glucose changes between the vascular and interstitial compartments of up to 5–6 minutes [74]. This implies that the significant negative cross-correlation between sleep spindles and changes in glucose concentration we observed at a time lag of 1 minute could well reflect that blood glucose begins to decrease prior to, rather than following, the detected sleep spindle. More generally speaking, this finding challenges the view that the spindle itself drives the decrease in peripheral glucose concentrations, but rather points to another factor, like a decrease in noradrenergic LC activity, that precedes the spindle and may promote the conjoint occurrence of the spindle and the decrease in glucose concentration. In a similar vein, the subtle glucose fluctuations (<1 mg/dL) detected in our study, while potentially physiologically meaningful, highlight the need for even more sensitive and precise measurement techniques. However, more advanced technology for continuous glucose monitoring required to capture metabolic dynamics with higher resolution is currently not available. Finally, our correlational analyses cannot establish causal mechanisms, and future work employing interventions—such as targeted modulation of sleep oscillations or active manipulation of glucose levels—will be needed to better understand the pathways underlying the associations reported here.

In summary, we show a fine-tuned coupling of signatures of memory processing during sleep, i.e. sleep spindles and SOs, to peripheral glucose concentrations in healthy humans. The changes after individual events are of small magnitude, averaging only a few mg/dL, and close to the sensitivity of our glucose monitoring system. Nevertheless, using event-related averaging to reduce noise in measurements, we confirmed that these changes were highly systematic and statistically significant. The small changes themselves after an individual sleep event, like a sleep spindle, are obviously without any clinical relevance. However, considering that these events occur repeatedly, hundreds of times during a single night, they may significantly contribute to the regulation and stabilization of glucose levels within the normal physiological range [25]. Considering also first clinical data showing strongly reduced spindle densities in patients with type 2 diabetes [75], investigations are warranted of whether and how the effects of spindles, SOs, arousals, and sleep stage transitions sum up acutely over the night to determine systemic glucose concentrations on the next day. Indeed, the associations we demonstrate may also be relevant in clinical conditions where sleep disruptions concur with perturbations of glucose regulation [6–8, 10, 12, 76, 77].

### Supplementary material

Supplementary material is available at *SLEEP* online.

## Supplementary Material

zsaf042_suppl_Supplementary_Table_S1_Figures_S1-S3

## Data Availability

The data underlying this article will be shared on reasonable request to the corresponding author.
