# Supplementary material for: Regulation of peripheral glucose levels during human sleep
Source: Sleep. 2025 Feb 23;48(6):zsaf042. doi: 10.1093/sleep/zsaf042 (PMC12163131; doi:10.1093/sleep/zsaf042)
Supplement: zsaf042_suppl_Supplementary_Table_S1_Figures_S1-S3 [file zsaf042_suppl_supplementary_table_s1_figures_s1-s3.docx]

**Regulation of peripheral glucose levels during human sleep**

**--- Supplementary Materials ---**

Xuefeng Yang^1,2^, Fernando Tavares Fedumenti^1^, Niels Niethard^1,3^, Manfred Hallschmid^1,4-6^, Jan Born^1, 4-7^ and Karsten Rauss^1^

^1^ Institute of Medical Psychology and Behavioral Neurobiology, University of Tübingen, Tübingen, Germany

^2^ Graduate School of Neural & Behavioural Science, International Max Planck Research School, Tübingen, Germany

^3^ Department of Cognitive Sciences, University of California, Irvine, Irvine, CA 92617, USA

^4^ German Center for Diabetes Research (DZD), Tübingen, Germany

^5^ Institute for Diabetes Research & Metabolic Diseases of the Helmholtz Center Munich at the University Tübingen (IDM), Tübingen, Germany

^6^ German Center for Mental Health (DZPG), Tübingen, Germany

^7^ Werner Reichert Center for Integrative Neuroscience, University of Tübingen, Tübingen, Germany

* Correspondence to: Karsten Rauss and Jan Born

karsten.rauss@uni-tuebingen.de or [jan.born@uni-tuebingen.de](mailto:jan.born@uni-tuebingen.de)

### *Sleep architecture*

Table S1. Sleep parameters for the individual nights

| **ID** | **D** | **TIB**  **(min)** | **TST**  **(min)** | **N1**  **(min)** | **N2**  **(min)** | **SWS**  **(min)** | **REM**  **(min)** | **SOL**  **(min)** | **WASO**  **(min)** | **Sleep**  **efficiency**  **(%)** | **Micro-**  **arousal**  **(counts)** |
| --- | --- | --- | --- | --- | --- | --- | --- | --- | --- | --- | --- |
| P1 | 1 | 474.5 | 382.0 | 61.0 | 248.0 | 7.5 | 65.5 | 14.5 | 49 | 80.51 | 18 |
|  | 2 | 491.0 | 464.0 | 53.5 | 238.0 | 74.0 | 98.5 | 4.5 | 21.5 | 94.50 | 52 |
| P2 | 1 | 472.0 | 446.5 | 49.5 | 241.0 | 61.0 | 95.0 | 6.5 | 19 | 94.60 | 42 |
|  | 2 | 512.0 | 478.0 | 49.0 | 256.0 | 67.5 | 105.5 | 6.0 | 27.5 | 93.36 | 26 |
| P3 | 1 | 479.5 | 433.0 | 43.0 | 249.5 | 53.5 | 87.0 | 10.5 | 36 | 90.30 | 53 |
|  | 2 | 481.0 | 418.0 | 55.0 | 186.0 | 105.5 | 71.5 | 12.0 | 24.5 | 86.90 | 17 |
| P4 | 1 | 479.5 | 299.0 | 55.0 | 116.5 | 41.5 | 86.0 | 14.5 | 162.5 | 62.36 | 49 |
|  | 2 | 480.0 | 405.0 | 49.5 | 212.5 | 57.0 | 86.0 | 7.5 | 59.5 | 84.38 | 7 |
| P5 | 1 | 480.5 | 404.0 | 42.0 | 231.0 | 54.0 | 77.0 | 12.5 | 62 | 84.08 | 50 |
|  | 2 | 480.0 | 446.5 | 38.0 | 224.0 | 82.5 | 102.0 | 14.0 | 13.5 | 93.02 | 50 |
| P6 | 1 | 483.0 | 400.5 | 39.5 | 230.5 | 32.0 | 98.5 | 13.0 | 37.5 | 82.92 | 22 |
|  | 2 | 480.5 | 420.0 | 69.0 | 274.5 | 0.5 | 76.0 | 28.5 | 32 | 87.41 | 29 |
| P7 | 1 | 475.0 | 427.0 | 39.0 | 271.0 | 39.5 | 77.5 | 8.0 | 40 | 89.89 | 5 |
|  | 2 | 483.5 | 450.0 | 52.0 | 253.5 | 50.0 | 94.5 | 1.5 | 21 | 93.07 | 42 |
| P8 | 1 | 481.0 | 307.0 | 23.0 | 160.0 | 51.0 | 73.0 | 26.0 | 148 | 63.83 | 22 |
|  | 2 | 483.0 | 424.5 | 33.0 | 220.5 | 61.5 | 109.5 | 16.0 | 42 | 87.89 | 10 |
| P9 | 1 | 482.0 | 430.0 | 23.5 | 209.5 | 66.0 | 131.0 | 14.0 | 36 | 89.21 | 33 |
|  | 2 | 481.5 | 429.0 | 38.5 | 231.0 | 54.0 | 105.5 | 4.0 | 48.5 | 89.10 | 44 |
| P10 | 1 | 483.5 | 468.5 | 22.0 | 256.0 | 61.5 | 129.0 | 3.5 | 11.5 | 96.90 | 79 |
|  | 2 | 484.5 | 458.5 | 25.5 | 273.0 | 63.5 | 96.5 | 4.0 | 22 | 94.63 | 92 |
| Mean |  | 482.38 | 419.55 | 43.03 | 229.10 | 54.18 | 93.25 | 11.05 | 45.68 | 86.94 | 37 |
| SEM |  | 1.79 | 10.46 | 2.95 | 8.71 | 5.22 | 3.98 | 1.59 | 8.96 | 2.07 | 5.11 |

*Note: Sleep parameters for the two nights of each participant. The respective first nights of participants P4 and P8 were excluded due to low sleep efficiency. Means and standard error of the mean (SEM) are reported without the excluded nights. All measurements are expressed in minutes. D: Day. TIB: Time in bed. TST: Total sleep time, time spent in N1, N2, SWS and REM sleep (in min). SOL: Sleep onset latency. WASO: Wake after sleep onset.SE: Sleep efficiency (TST / TIB * 100 %).*

### *Infraslow activity of sigma band activity and SWA during NonREM sleep*

Infraslow oscillations (ISOs) in the 0.01-0.1 Hz range were determined for power in the sigma band (10-15 Hz) and for slow-wave activity (SWA: 0.5 – 4 Hz) in NonREM sleep epoch > 120 s, using a procedure described previously ^1,2^. In brief, EEG data were segmented into 4-second intervals with 50% overlap, and after detrending and Hanning tapering, the absolute power spectral density was calculated for each segment. The spectral profile obtained for each night and channel was then normalized by dividing it by its mean power. Sigma and SWA power, respectively, were then subjected to spectral analysis to identify oscillatory frequency components. At each frequency, contiguous time sequences of at least 128 power component values were analyzed using Welch's periodogram method with 128-point windows, 75% overlap, and mean detrending. The final infraslow power spectrum was derived as the length-weighted average of the normalized spectral densities obtained from various contiguous sequences ^3^. To determine the peaks (and standard deviations) in the spectra, a Gaussian fit (four terms) was applied to the normalized power spectra from individual nights.


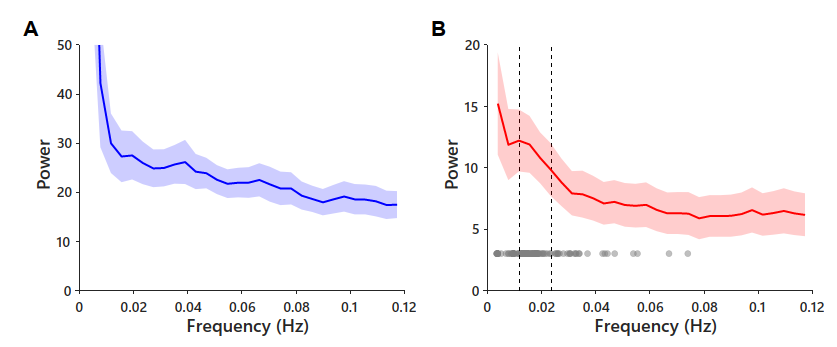


***Figure S1****. Average power spectrum of the time series* ***(A)*** *in SWA band power and* ***(B)*** *sigma-band power across all nights and recording channels. Dots in B denote peaks obtained in the individual spectra (using Gaussian fits). Vertical dashed lines indicate mean peak frequency ± 0.5 SD interval, highlighting a 0.02-Hz oscillation in sigma power. No distinct infraslow oscillation was observed in the time series of SWA power.*

### *Glucose levels decrease after isolated sleep spindles and increase after isolated SO*

Cross-correlation functions between the density of isolated spindles (i.e., spindle events not coupled to an SO) and glucose concentration changes showed a negative peak correlation at a time lag of +2 min (r = -0.06 ± 0.02; t(17) = -3.24, p = 0.005, BH-corrected p = 0.192; Figure S2A), indicating that an increased spindle density was followed, with a delay of 2 min, by a decrease in glucose. The decrease in glucose concentrations was likewise observed in the peri-event correlation histograms, emerging 3-7 min after the onset of an isolated spindle event (-0.62 ± 0.01 mg/dL in the 3-7 min interval; t(17) < -2.11, p < 0.05; Figure S2B). Increases in the density of isolated SO events, i.e., SOs not coupled with a spindle, predicted an increase in glucose 5 min later (r = 0.05 ± 0.02; t(17) = 2.31, p = 0.034, BH-corrected p = 0.20; Figure S2C). However, in the peri-event correlation histogram, this increase in glucose levels following isolated SO events failed to reach significance (Figure S2D).


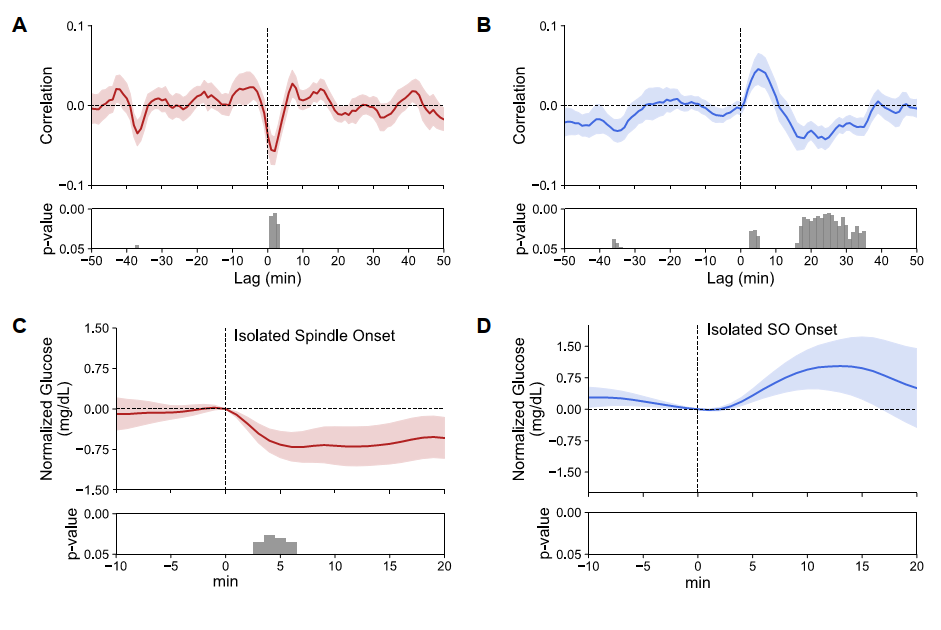


***Figure S2.*** *Changes in glucose concentration after spindles and SOs that occur in isolation, i.e., not coupled to an SO and spindle event, respectively.* ***(A)*** *Average cross-correlogram between glucose concentration change per min and density of isolated spindles and* ***(B)*** *isolated SO events, revealing negative and positive peak correlations at lags around +2 min and +5 min, respectively.* ***(C)*** *Peri-event time histogram showing the average time course of glucose concentration -10 to +20 min around the onset of isolated spindle events and* ***(D)*** *isolated SO events across all electrode sites and nights (n = 18, glucose level at 0 min = 90.61 ± 0.05 and 91.56 ± 0.04 mg/dL, respectively). Glucose concentrations are indicated as difference values with the concentration at 0 min set to 0 mg/dL Bottom panels indicate significance (one-sample t-test against zero).*

### *Activity in different frequency bands predicts distinct glucose concentration changes*

In supplementary analyses, we examined the cross-correlation functions between changes in glucose levels and power in specific EEG frequency bands hallmarking sleep, i.e., slow wave activity (SWA, 0.5-4 Hz), sigma band (10-15 Hz), and theta activity (4-8 Hz), for the entire recording period. Spectral analyses were performed using custom MATLAB scripts on subsequent 1-min segments on the filtered (0.5-30 Hz) EEG for each EEG channel (mtmfft, FieldTrip) ^4,5^. Power for the frequency bands of interest was expressed as a proportion of the total broadband power (0.5-30 Hz). Statistical analyses were performed on the channel showing the highest power for the respective band, i.e., Fz was used for SWA and the theta band, and Pz for the sigma band. Results for SWA and sigma power were consistent with the findings for SOs and spindles, respectively, reported in the main text (Figure S3A, B). Results for theta activity fitted with those for REM sleep transitions, with an increase in theta activity predicting a decline in glucose levels 2 min later (r = -0.12 ± 0.02, temporal lag = 2 min; t(17) = -4.75, p < 0.001, BH-corrected p = 0.003; Figure S3C).


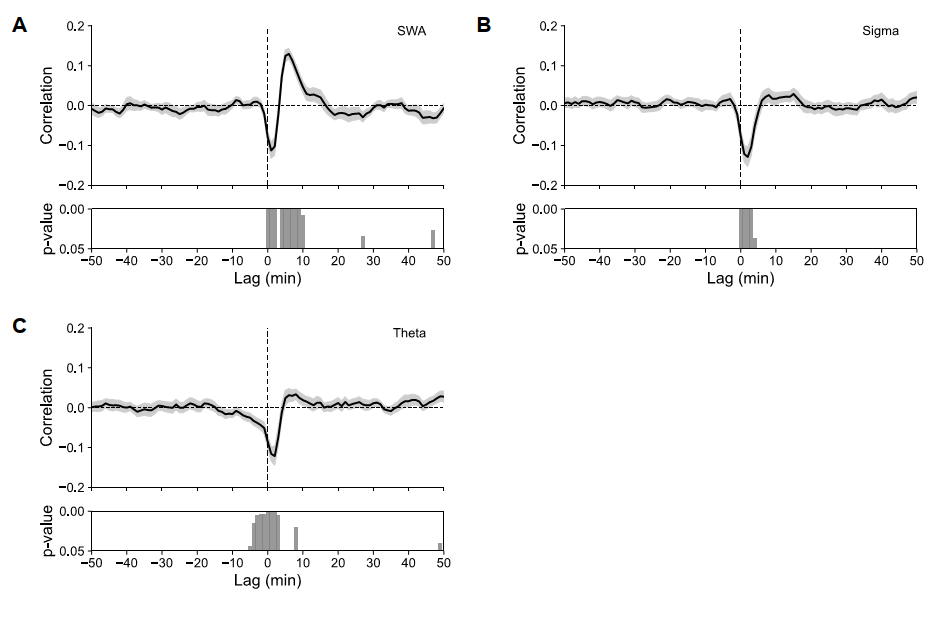


***Figure S3****. Cross-correlation functions between power in specific EEG frequency bands and changes in glucose concentrations.* ***(A)*** *Average cross-correlogram between glucose concentration change per min and power in the SWA (0.5-4 Hz),* ***(B)*** *sigma (10-15) and* ***(C)*** *theta (4-8 Hz) frequency bands. For positive lags changes in EEG power precede changes in glucose concentration. Bottom panels indicate significance of correlation coefficients (one-sampled t-test against 0).*

## References

1. Cardis R, Lecci S, Fernandez LMJ, et al. Cortico-autonomic local arousals and heightened somatosensory arousability during NREMS of mice in neuropathic pain. Kuner R, Büchel C, Saab CY, eds. *Elife*. 2021;10:e65835. doi:10.7554/eLife.65835

2. Lecci S, Fernandez LMJ, Weber FD, et al. Coordinated infraslow neural and cardiac oscillations mark fragility and offline periods in mammalian sleep. *Sci Adv*. 2017;3(2):e1602026. doi:10.1126/sciadv.1602026

3. Lázár ZI, Dijk DJ, Lázár AS. Infraslow oscillations in human sleep spindle activity. *J Neurosci Methods*. 2019;316:22-34. doi:10.1016/j.jneumeth.2018.12.002

4. Oostenveld R, Fries P, Maris E, Schoffelen JM. FieldTrip: Open Source Software for Advanced Analysis of MEG, EEG, and Invasive Electrophysiological Data. *Comput Intell Neurosci*. 2011;2011:1-9. doi:10.1155/2011/156869

5. Prerau MJ, Brown RE, Bianchi MT, Ellenbogen JM, Purdon PL. Sleep Neurophysiological Dynamics Through the Lens of Multitaper Spectral Analysis. *Physiology*. 2017;32(1):60-92. doi:10.1152/physiol.00062.2015
